# Change in WHO cardiovascular risk prediction over three years in PLWH on efavirenz-based ART

**DOI:** 10.4102/sajhivmed.v26i1.1697

**Published:** 2025-07-16

**Authors:** Melani Ratih Mahanani, Ethel Rambiki, Tapiwa Kumwenda, Claudia Wallrauch, Tom Heller, Volker Winkler, Florian Neuhann, Hans-Michael Steffen

**Affiliations:** 1 Heidelberg Institute of Global Health, University of Heidelberg, Heidelberg, Germany; 2 Centre for Preventive Medicine and Digital Health, Division of Prevention of Cardiovascular and Metabolic Conditions, Medical Faculty Mannheim, University of Heidelberg, Mannheim, Germany; 3 Lighthouse Clinic, Kamuzu Central Hospital, Lilongwe, Malawi; 4 Institute of Infectious Diseases and Tropical Medicine, LMU University Hospital, Ludwig-Maximilians University of Munich, Munich, Germany; 5 International Training and Education Center for Health, University of Washington, Seattle, United States of America; 6 School of Medicine and Clinical Sciences, Levy Mwanawasa Medical University, Lusaka, Zambia; 7 Department of Gastroenterology and Hepatology, Faculty of Medicine, University Hospital Cologne, University of Cologne, Cologne, Germany; 8 Hypertension Center, Faculty of Medicine, University Hospital Cologne, University of Cologne, Cologne, Germany; 9 Department of Postgraduate Studies and Research, Chreso University, Lusaka, Zambia

Despite the widespread use of effective antiretroviral therapy (ART), sub-Saharan Africa still suffers from the highest global share of incident HIV cases (64.8%), AIDS deaths (74.0%), and persons living with HIV (PLWH, 70.7%).^1^ While HIV infection itself must be considered as a cardiovascular disease (CVD) risk factor,^2^ it is estimated that 20% to 40% of PLWH in sub-Saharan Africa have hypertension,^3^ with ensuing higher rate of cardiovascular events and increased all-cause mortality in comparison to hypertensive HIV-uninfected individuals or normotensive PLWH.^2^ Meta-analyses estimated that the prevalence of hypertension is between 14.5% and 35.0% for PLWH on ART, compared to 10.5% to 16.9% for ART-naïve PLWH.^4,5,6^

Currently, formal assessments of cardiovascular risk in Malawian PLWH are only available from three cross-sectional studies.^7,8,9^ We have recently published our observations on the prevalence, incidence, and control of hypertension and the determinants of blood pressure (BP) changes in the prospective LighTen Cohort study.^10^ Here we present data on the change in cardiovascular risk over 3 years assessed with the WHO CVD risk prediction charts^11^ in PLWH who were followed for up to 3 years after initiation of tenofovir-based ART in Lilongwe, Malawi.

The study was conducted at Lighthouse, a WHO-recognised Centre of Excellence for integrated HIV services located at Kamuzu Central Hospital, the tertiary hospital in central Malawi. About 12 000 PLWH received care at the clinic during the study period. The LighTen study involved adult (≥ 18 years) PLWH who presented to the facility for the initiation of ART and who started a fixed-dose combination of 300 mg tenofovir disoproxil fumarate (TDF), 300 mg lamivudine (3TC), and 600 mg efavirenz (EFV) in accordance with Malawi HIV treatment guidelines at the time. In addition to being collected at baseline (month 0), patient history, current medication, and anthropometric data were also taken during routine study visits at 1, 3, 6 months, and then every 6 months until month 36.^10^

Every visit included at least two oscillometric office BP measurements (Omron M300 [Omron Corporation, Japan], or Rossmax CF115f [Rossmax International Ltd., Taiwan] in the attendance of health care personnel) and their mean was then classified in accordance with the 2020 International Society of Hypertension Global Hypertension Practice Guidelines. Throughout the first three visits (month 0, 1, and 3), a mean BP of less than < 140/90 mmHg per visit on at least two occasions was considered as normotension. As a mean BP ≥ 140/90 mmHg on one occasion does not identify a patient as ‘hypertensive’, the individual median or modal BP category of the first three visits was considered as each participant’s hypertension category at baseline. PLWH, categorised that way as ‘hypertension stage 1’ or higher plus participants receiving antihypertensive therapy, were considered as prevalent hypertension. Mean BP of at least 140/90 mmHg on two consecutive visits starting at month 6 was considered as incident hypertension. The antihypertensive medication treatment was provided at no cost and was prescribed in a stepwise manner according to the Malawi Standard Treatment Guideline.

The non-laboratory-based WHO Risk Prediction chart for eastern sub-Saharan Africa was used to estimate the 10-year risk of a major CVD event for participants aged 40 to 74 years, as cholesterol measurements were not available.^11^ Since this chart is not applicable in case of diabetes, the 2007 WHO / International Society of Hypertension (ISH) Cardiovascular Risk Prediction Charts for WHO sub-region AFR E was used for three drug-treated (metformin) patients with type 2 diabetes mellitus (T2DM). The risk was visualised graphically from the baseline to the last visit using a Sankey diagram. Frequencies were presented for categorical variables and medians with interquartile ranges for continuous variables. Mixed effects ordered logistic regression was used for categorical variables and the Friedman test for continuous variables to determine if there is a difference between visits at months 0, 18 and 36. Stata/IC 15.1 (StataCorp, College Station, Texas, United States) and SPSS 29.0.2.0 (IBM Corp, Armonk, New York, United States) were used to perform the statistical analyses. Two-tailed tests resulting in a *P*-value < 0.05 were deemed statistically significant.

At treatment initiation, 413 participants age ≥ 40 years were assessed. At baseline, 56 of 197 women (28.4%) and 60 of 216 men (27.8%) were identified as hypertensive. The prevalence of hypertension increased with age, ranging from 22.1% to 47.8% for those aged 40–44 years to ≥ 60 years. During the follow-up period 18 participants died, 42 were transferred out to other facilities, 11 withdrew their participation consent, 57 defaulted, 20 changed ART regimens, and one completely stopped ART. The change in CVD risk was analysed in 201 participants (108 men, 53.7%) who were followed up until month 36 and had complete data on BP and body mass index. Over 3 years, hypertension was newly detected in 8.5% of women and 7.1% men, and the incidence of hypertension increased with age, from 6.8% for PLWH 40–44 years old to 16.6% for PLWH ≥ 60 years. The number of PLWH receiving antihypertensive drug treatment increased from 15 at baseline to 37 at the final visit. However, BP was uncontrolled (≥ 140/90 mmHg) initially among 10 of the 15 participants (66.6%), and in 27 of 37 patients (73%) at month 36 (Table 1).

**TABLE 1:** Characteristics at antiretroviral therapy initiation and after 36 months on tenofovir disoproxil fumarate/lamivudine/efavirenz for 201 patients aged 40–74 years.

Characteristics	Baseline visit	18 months visit†	36 months visit	*P*
Age in years‡	45 (8)	47 (9)	48 (8)	< 0.001
Weight in kg‡	59.9 (15.6)	61.9 (15.2)	64 (16.6)	< 0.001
BMI in kg/m^2^;‡	23.8 (6.4)	24.7 (6.3)	25.4 (7.2)	< 0.001
**BMI classification in kg/m^2^;**	-	-	-	0.020
< 18.5§	6	4	2	-
18.5–24.9§	114	99	93	-
25.0–29.9§	48	58	56	-
≥ 30.0§	33	37	50	-
**ISH classification**	-	-	-	0.834
Normal¶	121 (3)	118 (7)	119 (7)	-
High-normal¶	27 (2)	39 (4)	34 (3)	-
Hypertension stage 1¶	32 (4)	30 (13)	35 (18)	-
Hypertension stage 2¶	21 (6)	10 (8)	13 (9)	-

BMI, body mass index; ISH, International Society of Hypertension; IQR, interquartile range.

† , visit 18 months only available for *n* = 198, blood pressure documented for *n* = 197 of that visit;

‡ , median (IQR);

§ , number of patients within the specified BMI classification;

¶ , *N* (*n*): number of patients within the specified ISH classification, (*n*) number of patients receiving antihypertensive drug treatment.

**FIGURE 1:**
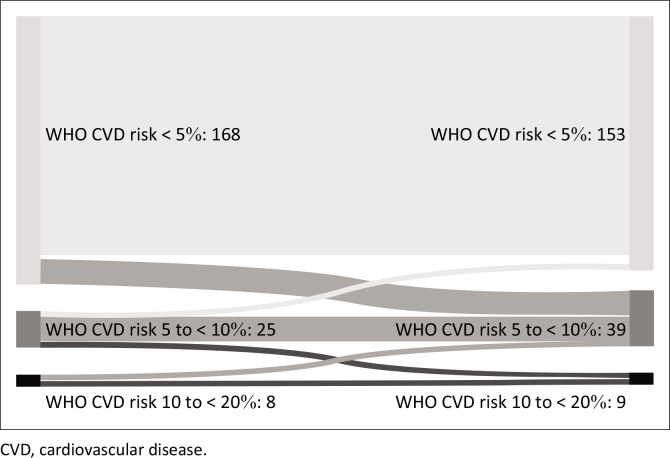
Change in WHO cardiovascular disease risk categories over 36 months on antiretroviral therapy for individuals aged 40–74 years. Comparison between baseline and 36-month measurements: *P* = 0.073. A change in risk from 5% to < 10% to 10% to < 20% or vice versa was observed in four and three cases, respectively.

There were only three participants with T2DM (all metformin-treated), and their initial CVD risk was translated from 2007 WHO/ISH Cardiovascular Risk Prediction Charts to the 5% to < 10% (*n* = 2) and 10% to < 20% (*n* = 1) category, respectively. Overall, CVD risk increased by one category in 24 patients (11.9%) and decreased in 8 (4.0%). The increase in CVD risk followed either weight gain (*n* = 5), an increase in BP (*n* = 4) or both (*n* = 12). Age was the sole responsible factor for increasing risk in three cases. The highest rate of change (*n* = 20) in WHO CVD risk category was found for participants with an initial risk < 5% (Figure 1).

Using the validated WHO CVD Risk Prediction Charts for East Africa, the patterns observed may still have clinical relevance, although statistical significance was not achieved. Small but consistent shifts in CVD risk categories could indicate meaningful long-term trends that warrant further investigation. Further, this increase was associated with weight gain and uncontrolled hypertension, despite higher rates of antihypertensive treatment and regular outpatient visits.

The prevalence of diabetes in PLWH from earlier studies ranged from 4.1% to 6.6% and is higher than the 1.5% (*n* = 3) in our current study, where the diagnosis relied only on known antidiabetic drug treatment. Data from studies with available lipid measurements calculated risks ≥ 20% in 2.4% overall^9^ and up to 23.7% for those aged 60 years or older,^8^ according to the Framingham risk score.^8,9^ However, this risk score has never been validated for African populations.

When interpreting our data, we consider several limitations. First, there was a considerable number of patients lost to follow-up in our cohort, which subjects the results to attrition bias and affects the power of this study, limiting the ability to detect a significant difference. Second, the duration of our study was too short to allow analyses of more robust outcomes, such as cardiovascular cause-specific death and morbidity. Third, the 2019 WHO laboratory-based CVD risk prediction charts would have been more precise in risk estimation than the non-laboratory-based charts, especially with respect to patients with T2DM. Fourth, dyslipidaemia was not assessed and, together with the low number of study participants identified and analysed as having T2DM, this can explain why there were no cases of a cardiovascular risk > 20%. Fifth, the risk of atherosclerotic cardiovascular disease is twice as high among PLWH compared to the general population, even in younger people and those at lower traditional risk for CVD.^2,12^ Since the WHO risk prediction charts^11^ rely on traditional risk factors, the true CVD risk burden in our study population and its change over time may have been underestimated. Finally, considering the greater weight gain associated with first-line use of integrase strand transfer inhibitors, for example dolutegravir, our findings do not apply to these newer ART regimens.^13,14^ In conclusion, formal assessment of cardiovascular risk using the validated WHO scoring system suggests weight gain and uncontrolled hypertension despite higher treatment rates as important drivers behind increased CVD risk in PLWH on ART. Our short-term observation over only 3 years confirms the need for integrated noncommunicable disease health services at HIV treatment centres in low- and middle-income countries such as Malawi.^15^
